# Supplementary material for: Damage-induced reactive oxygen species enable zebrafish tail regeneration by repositioning of Hedgehog expressing cells
Source: Nat Commun. 2018 Oct 1;9:4010. doi: 10.1038/s41467-018-06460-2 (PMC6167316; doi:10.1038/s41467-018-06460-2)
Supplement: Supplementary file 1 — Supplementary Information [file 41467_2018_6460_MOESM1_ESM.pdf]

SUPPLEMENTARY INFORMATION FILE

Damage-induced reactive oxygen species enable regenerative signalling by the rapid repositioning of Hedgehog expressing cells.

Romero *et al.*

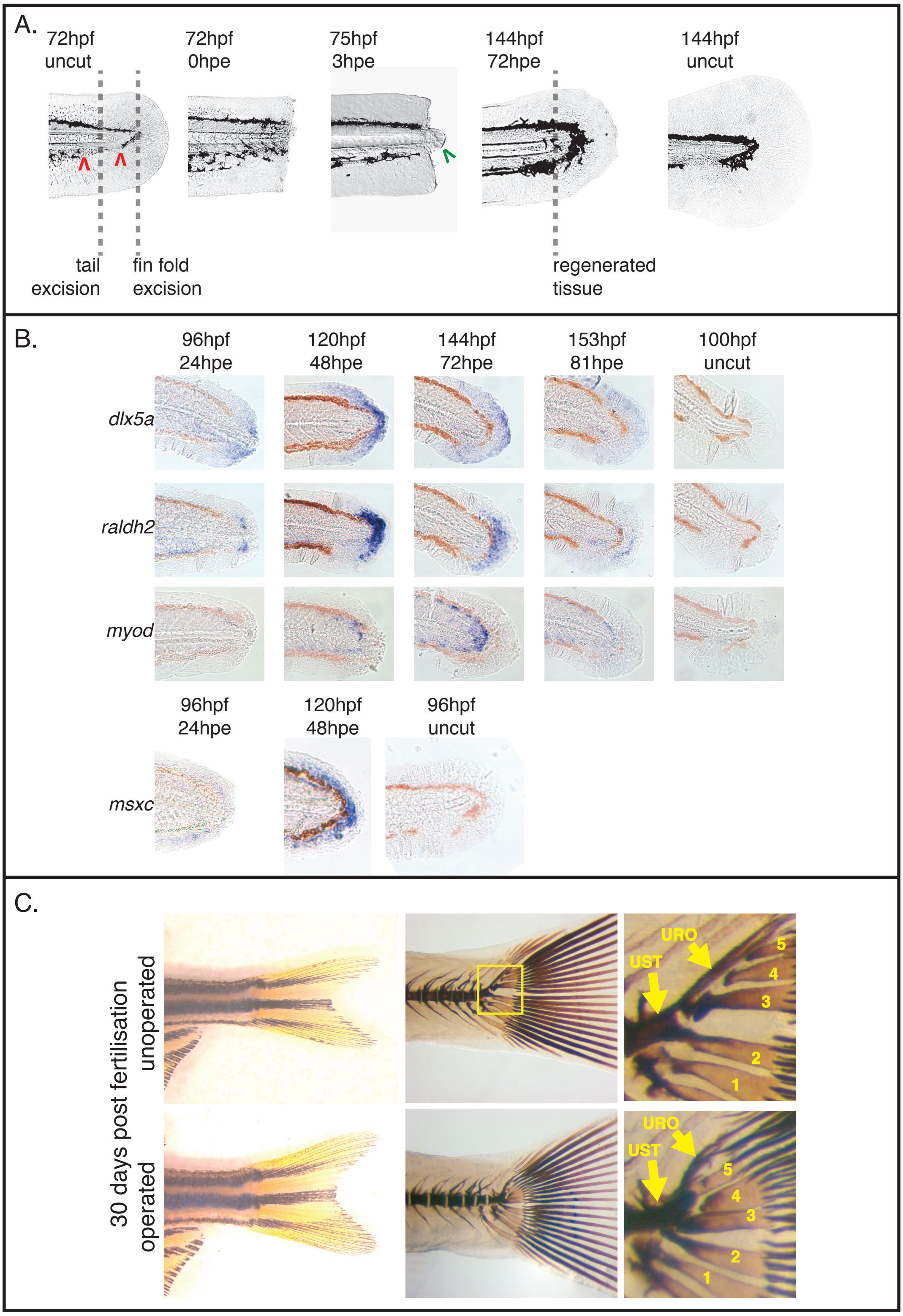

Supplementary Figure1. Larval tail regeneration. (A) When cut in the pigment gap (zone between the two red arrows), the notochord bead (the green arrow) is extruded within 3hpe and the tail regenerates within 4-5 days. The position of fin fold excision is shown for comparison. (B) Different stages of regeneration are marked by the expression of *dlx5a*, *raldh2*, *myod* and *msxc* (all panels 10/10 except *dlx5a*/24hpe 8/10, *raldh2*/24hpe 8/10, *myod*/48hpe 8/10, number of experiments = 3). Uncut fish are shown to the right for comparison. (C) Live images of the tail after metamorphosis is complete (left panels) and Von Kossa stain showing the failure of the notochord to extend dorsally (right panels). In unoperated fish the uroneural and hypurals 3-5 form around and are attached to the notochord which grows dorsally as the adult tail forms. After excision these bones all form on the urostyle suggesting that the dorsal flexure of the notochord has not occurred (both operated and unoperated are 5/5, number of experiments = 2). Abbreviations: urostyle (ust), uroneural (uro) and hypural 1-5 (1-5).

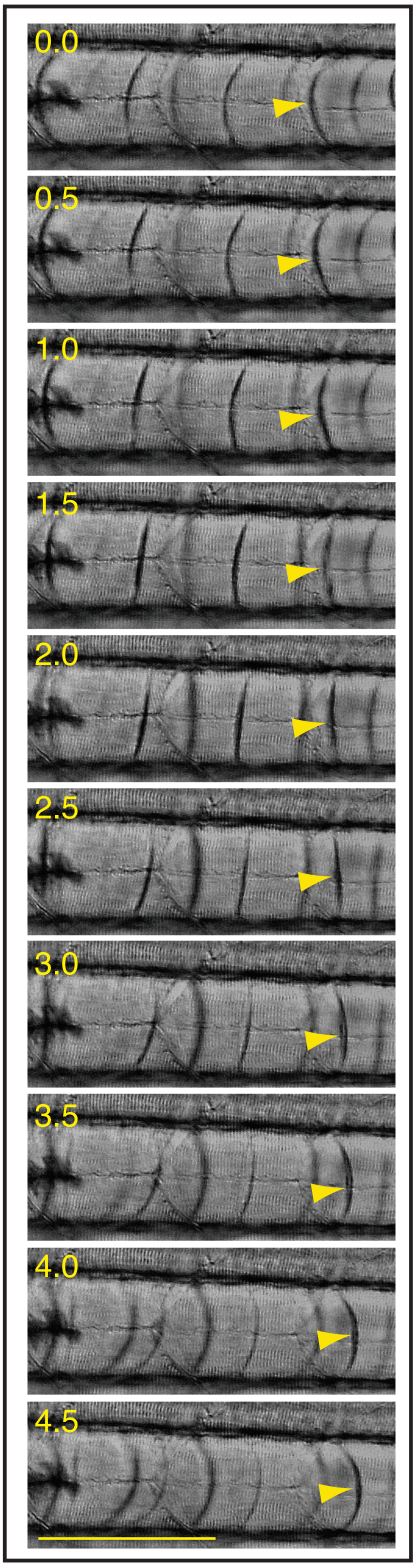

Supplementary Figure 2. There appears to be a change in pressure within the notochord at the start of extrusion. Close-up of notochord after tail excision to show cell membranes are bowed towards anterior at the start and then become bowed towards the posterior. The yellow arrowhead tracks one membrane. Not all fish show bowing to the anterior, the prevalence of this phenotype varies by age, position on the anterior/posterior axis and strain used. We have found the AB wildtype strain at 72hpf is most reliable. Images captured every 30 seconds as in Supplementary Movie 1. Numbers indicate time in minutes. Scale bar = 100µm.

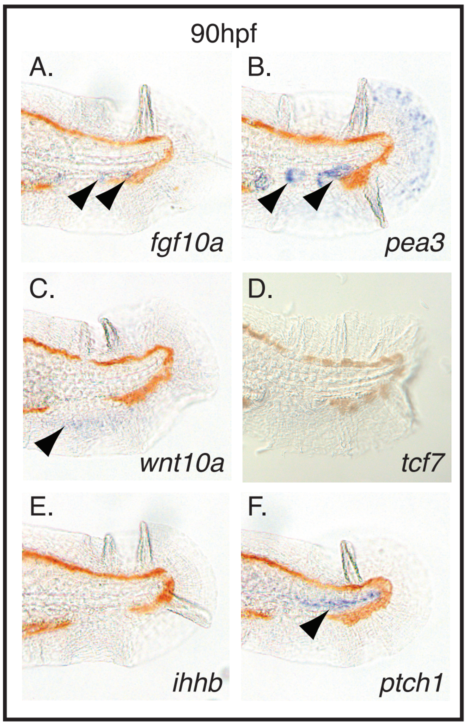

Supplementary Figure 3. Expression of signalling genes in the larval tail. (A) *fgf10a* is expressed weakly in neuromasts (arrowheads). (B) *pea3* is expressed in neuromasts (arrowheads) and has weak expression in the fin fold. (C) *wnt10a* has weak expression in the ventral fin fold (arrowhead). (D) *tcf7* has no detectable expression. (E) *ihhb* has no detectable expression. (F) *ptch1* has weak expression in tissue neighbouring the notochord (arrowhead) (10/10 for all panels, number of experiments = 3).

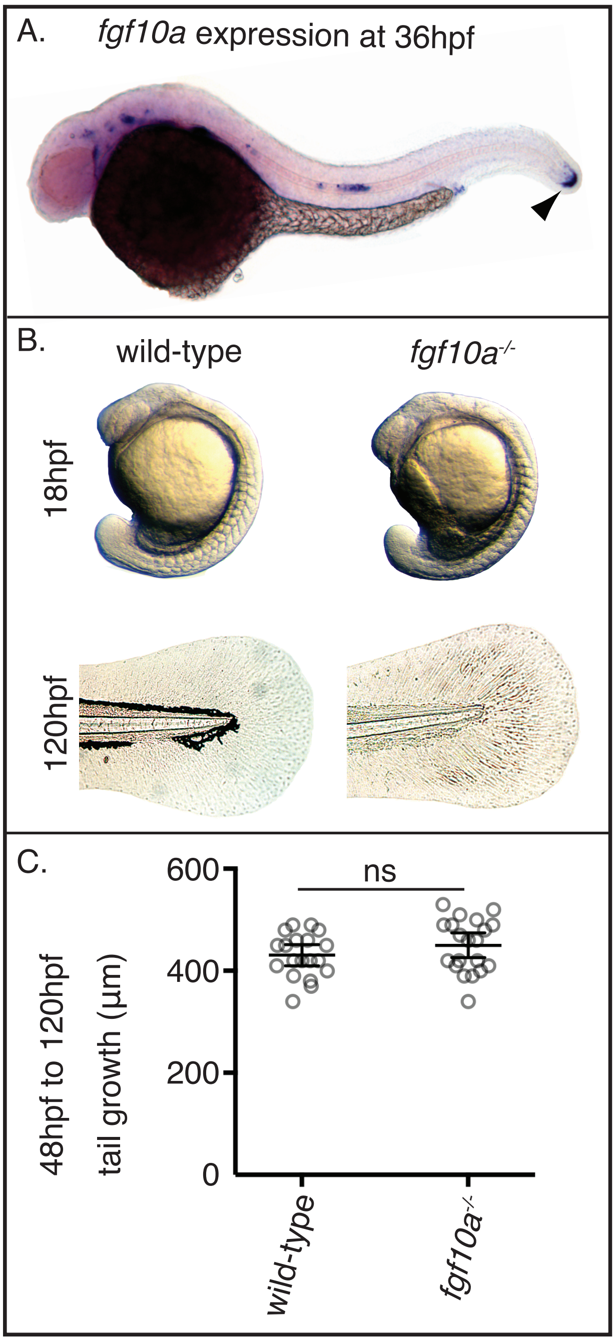

Supplementary Figure 4. *fgf10a* does not act during tail development. (A) Arrowhead indicates *fgf10a* expression in the tail. (B) Development of the *fgf10a*$^{-/-}$ larval tail is indistinguishable from wild-type. (C) Measurement of tail growth does not show a significant difference between *fgf10a*$^{-/-}$ and wild-type fish. Tails were measured as in Figure 1 (n = 20 for both, number of experiments = 2).

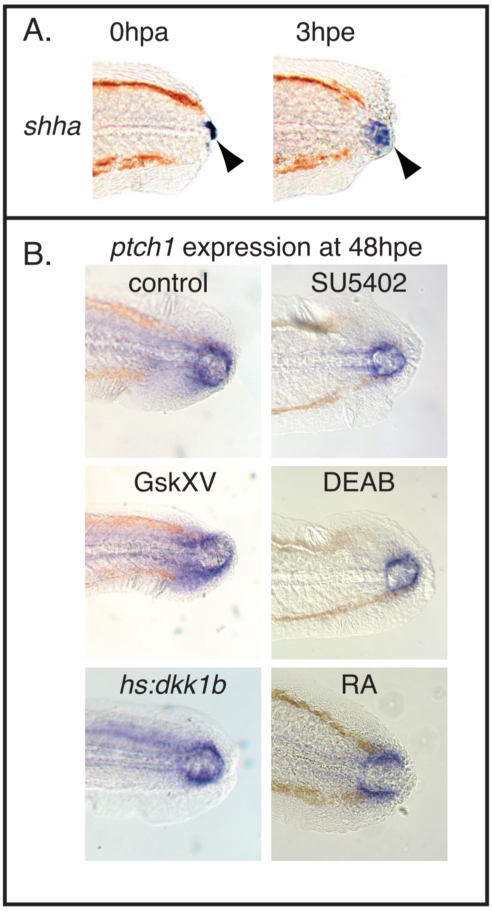

Supplementary Figure 5. *shha* is expressed in the notochord bead and Hedgehog signalling is unaffected by manipulation of other developmental pathways. (A) *shha* is expressed in the notochord bead (arrowheads) (n = 3/7 for 0hpe and 11/12 for 3hpe, number of experiments = 2). (B) The activity of the Hedgehog pathway was monitored by in situ hybridisation for *ptch1*. Larvae were treated with GskXV at 12.5μM from 40-48hpa to activate the WNT/β-Catenin pathway. *hs:dkk1b-GFP* fish were heatshocked for three hours at 39°C at 28hpa and allowed to recover for 17 hours at 28.5°C to inhibit the Wnt/β-Catenin pathway. Larvae were treated with SU5402 at 15μM from 44-48hpf to inhibit the FGF pathway. Larvae were treated with 100 μM DEAB from 1-48hpa to block the RA pathway. Larvae were treated with RA at 2.5μM from 43.5-48hpf to activate the RA pathway (10/10 for all panels, number of experiments = 2).

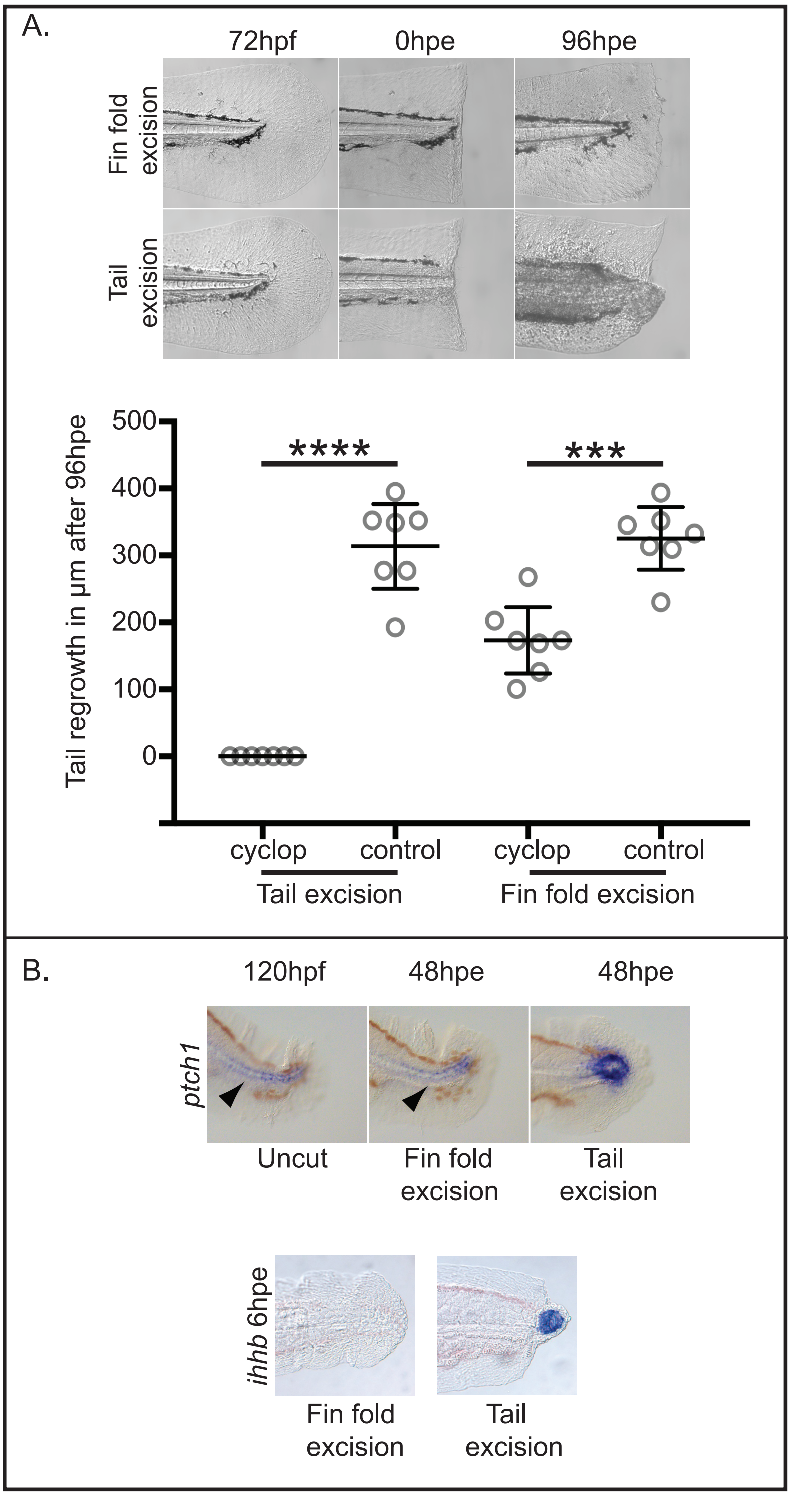

Supplementary Figure 6. Hedgehog signalling does not play a crucial role in fin fold regeneration. (A) Treatment with 20µM cyclopamine is sufficient to block regeneration and has a milder affect on fin fold excision. Regrowth was measured from images in Fiji/ImageJ software by placing a rectangle parallel to the body that started at the end of the notochord and finished at the caudal end of the fin fold (n = 7 for each sample, number of experiments = 2). (B) Neither *ptch1* nor *ihhb* are noticeably upregulated following fin fold excision. Arrowheads indicate low levels of *ptch1* expression in unoperated and fin fold excision larvae (10/10 for each sample except for *ihhb*/tail excision 9/10, number of experiments = 2).

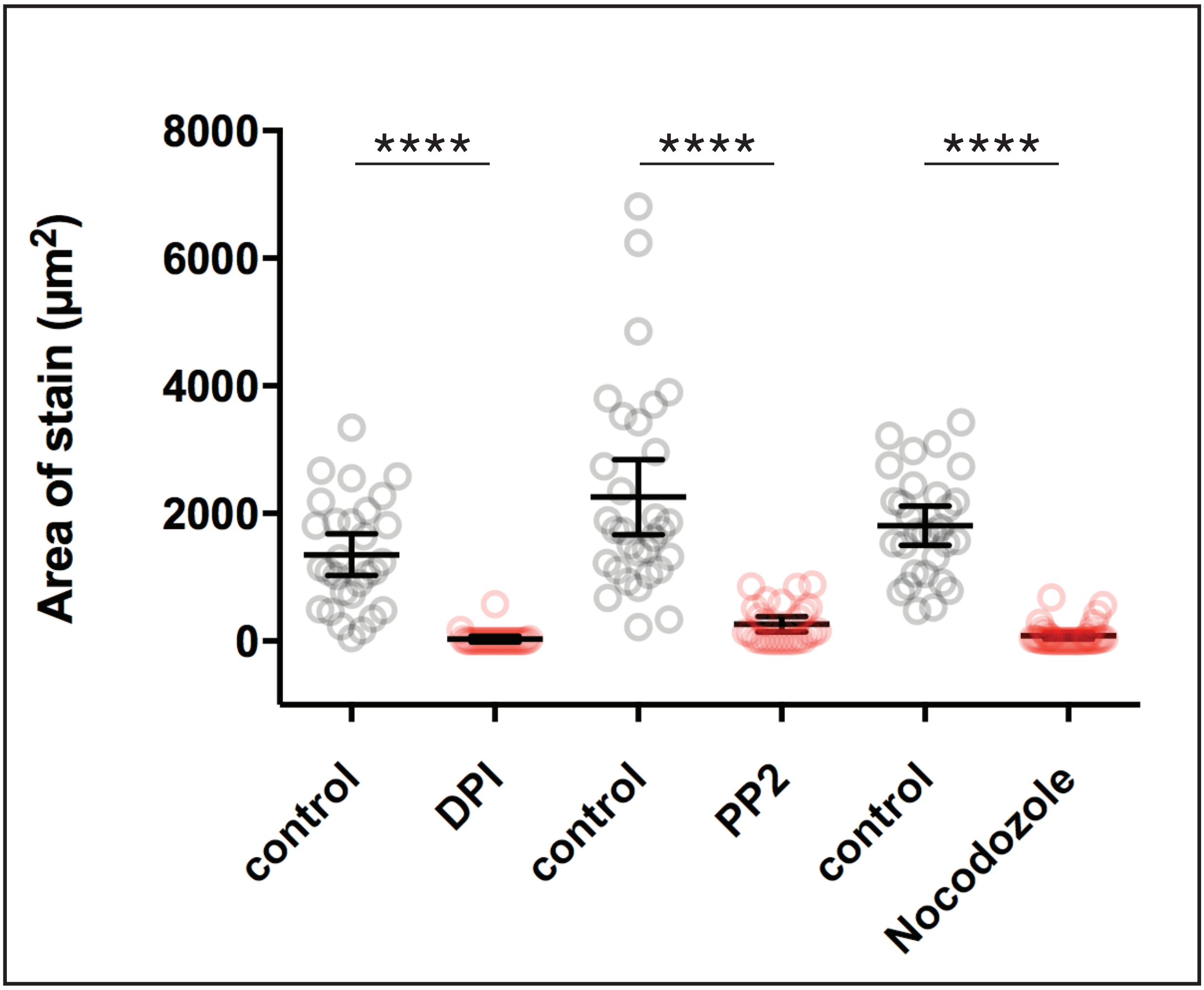

Supplementary Figure 7. Wound-induced *ptch1* expression is reduced at 24hpe after treatment with inhibitors. Quantification of staining shows that all treatments reduced the level of expression of *ptch1*. DPI (150μM) treatment started at 1 hour prior to wounding and ended at 1hpa, after which larvae were washed in E3 buffer and incubated until 6hpe. MCI186 (2mM) treatment started at 0hpe and ended at 3hpe. PP2 (20 μM) treatment started at 1 hour prior to wounding and ended at 6hpe. Nocodazole (10 μg/mL) treatment started at 0hpe. (DMSO n = 29, DPI n = 26, number of experiments = 2), (DMSO n = 31, PP2 n = 27, number of experiments = 2), (DMSO n = 30, nocodozol n = 38, N = 2). Statistics shown are unpaired t test two-tailed with **** indicating P < 0.0001.

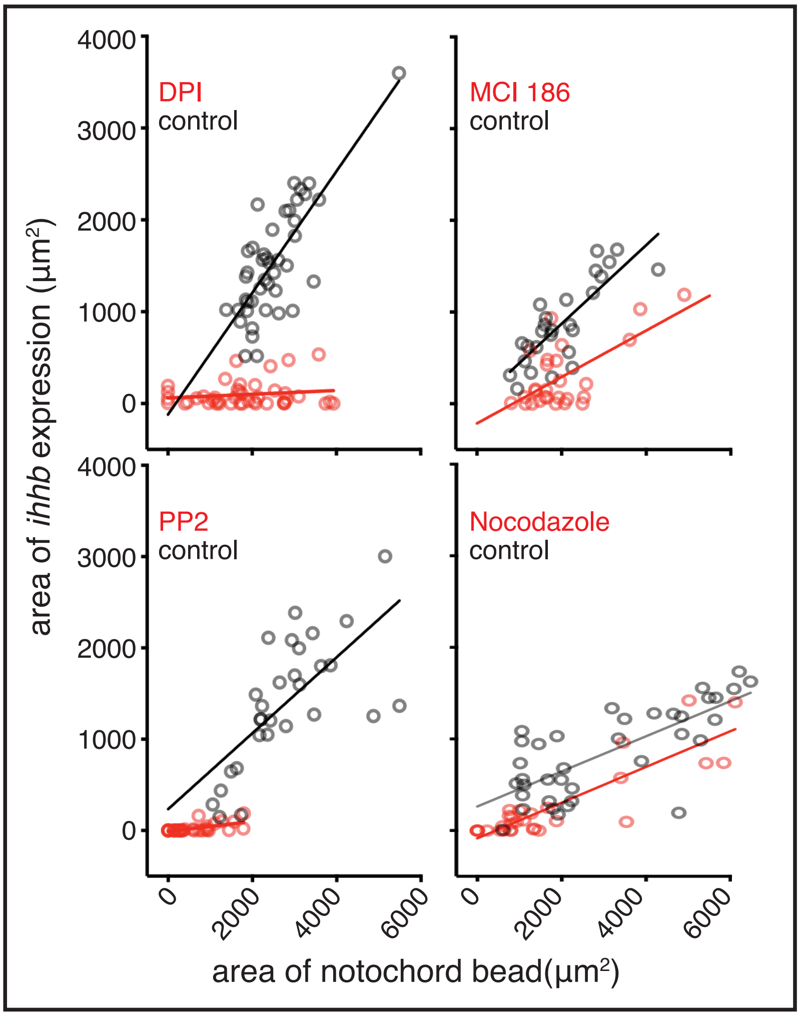

Supplementary Figure 8. Treatment with DPI, MCI 186, PP2 or nocodazole and comparison of bead size to expression of *ihhb*. Data from Fig. 6, panels a and c plotted together for direct comparison. Regression lines suggest that nocodazole has the strongest correlation and DPI has the weakest.

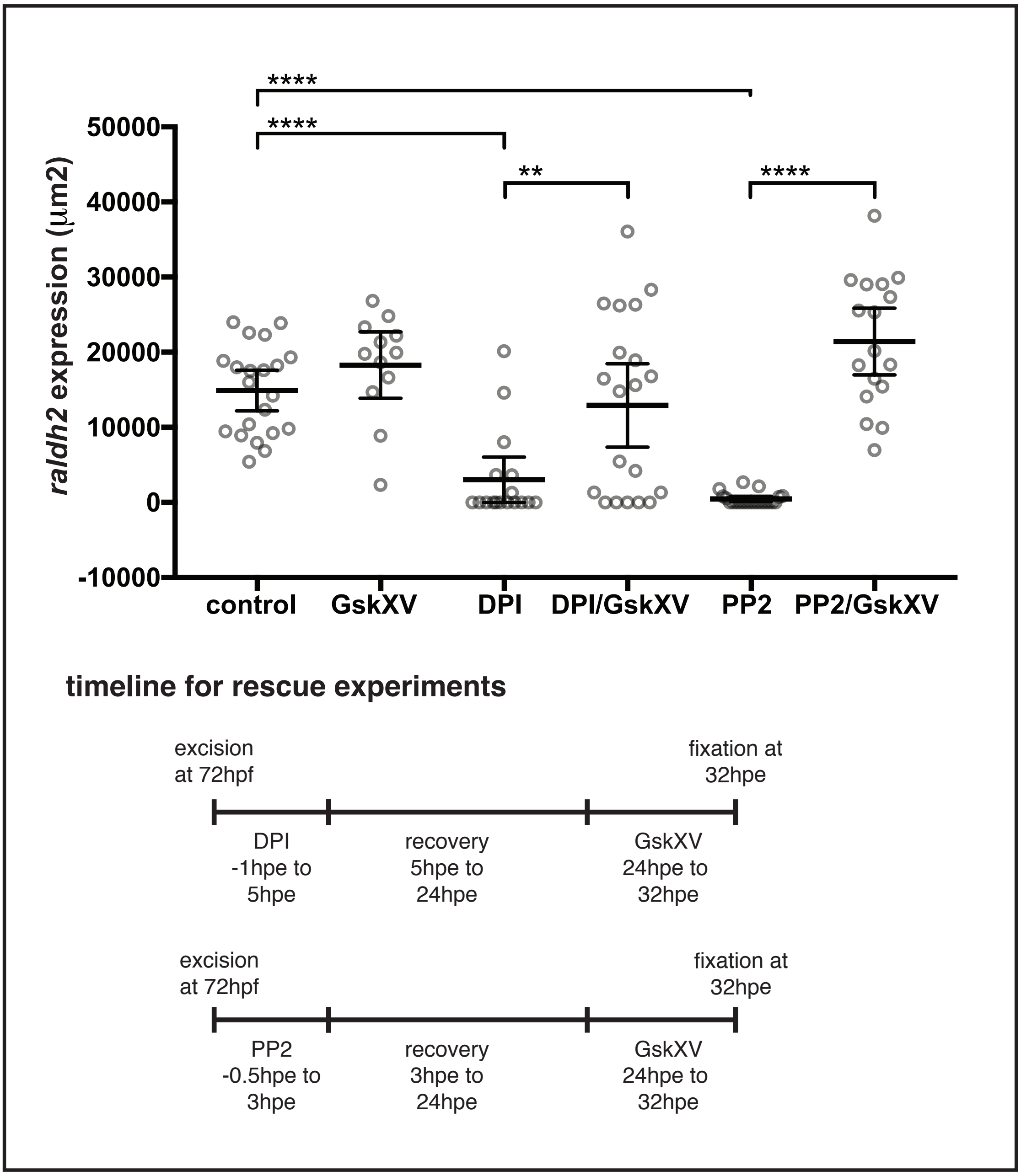

Supplementary Figure 9. Treatment with DPI and PP2 is partially rescued by upregulation of the Wnt/β-Catenin pathway. Larvae were operated on at 72hpf and treated with DPI, PP2 alone or in combination with GskXV. For DPI treatments, larvae were pre-treated for one hour prior to excision at 150μM concentration, then in 100μM for 5hpe. For PP2 treatments, larvae were pre-treated for thirty minutes prior to excision at 20μM concentration, then in 20μM for 3hpe. Larvae were then washed three times, incubated until 24hpe and treated with 10μM GskXV from 24hpe to 32hpe. Control fish were treated with DPI or PP2 alone (as above), or with the solvent DMSO at 1.5% continuously or with 10μM GskXV alone from 24 to 32hpe. Larvae were fixed at 32hpe and processed for RNA in situ analysis with *raldh2*. Significance was determined using ordinary one-way ANOVA (non-parametric) with multiple comparisons and Sidak hypothesis. (DMSO n = 21, GskXV n = 12, PP2 n = 21, DPI n = 17, PP2/GskXV n = 17, DPI/GskXV n = 20, number of experiments = 2). Significance of **** indicates P = 0.0001 and ** indicates 0.0015.

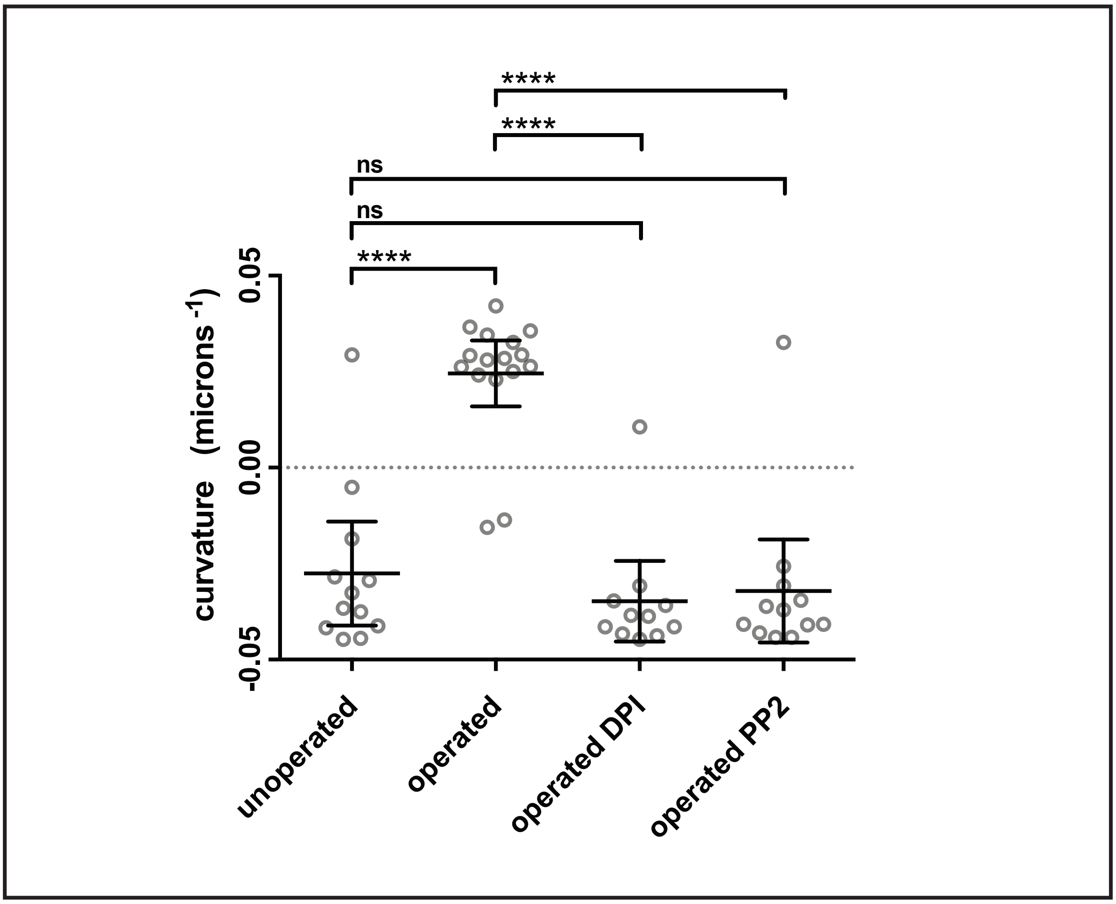

Supplementary Figure 10. Menger curvature measurements show that notochord cell shape changes are altered by treatment with DPI or PP2. Fish were pretreated for two hours with DPI (150μM) or PP2 (20μM) at 28.5°C. Then fish were operated and incubated at 21°C with imaging taking place at twenty minutes post excision. Control fish were treated with 1.5% DMSO. Significance was determined using ordinary one-way ANOVA (non-parametric) with multiple comparisons and Sidak hypothesis testing. (unoperated n = 12, operated n = 16, operated DPI n = 11, operated PP2 n = 12, number of experiments = 3). **** indicates $P < 0.0001$.

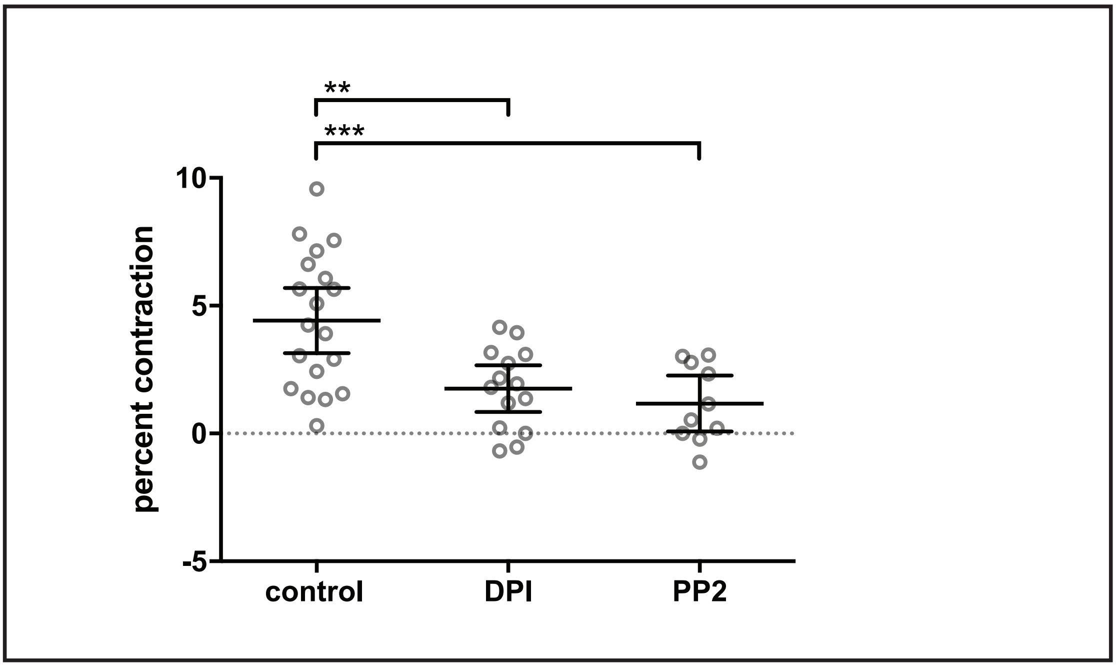

Supplementary Figure 11. Wound induced contraction of the trunk is reduced by treatment with DPI or PP2. Fish were pretreated for two hours with DPI (150µM) or PP2 (20µM). Then fish were imaged immediately prior to tail excision, incubated individually for a further two hours in DPI (150µM) or PP2 (20µM) and imaged again. Control fish were treated with 1.5% DMSO. Significance was determined using Ordinary One-Way ANOVA (non-parametric) with multiple comparisons and Sidak hypothesis testing. (Control n = 19, operated n = 16, DPI n = 14, operated PP2 n = 10, each fish was treated and measured separately so the number of experiments equals n). ** indicates $P < 0.0018$, *** indicates $P < 0.0007$.

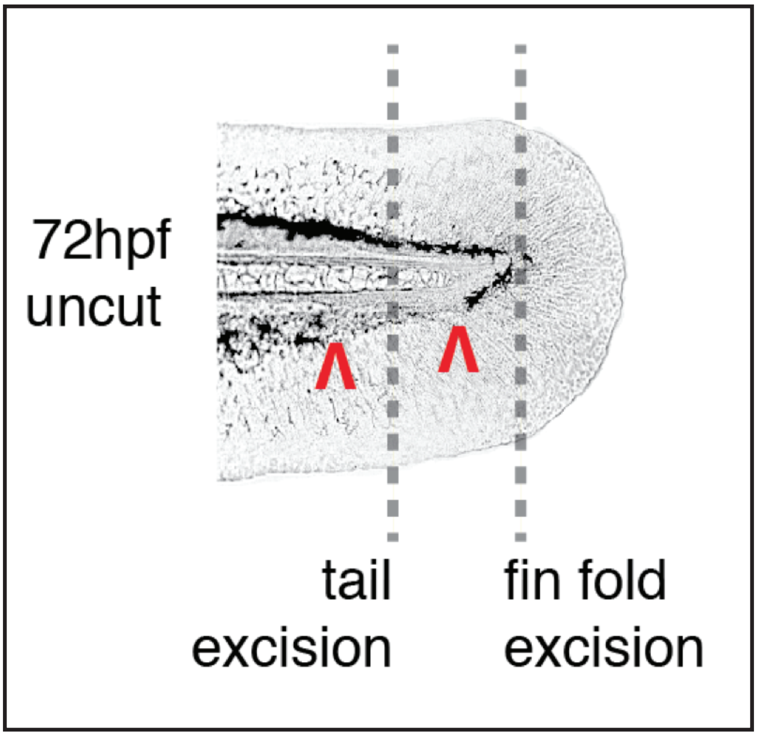

Supplementary Figure 12. Methods image to illustrate the position of tail excision. The picture shows the position for tail excision compared to fin excision (grey dotted lines). The pigment gap marked by red arrowheads.

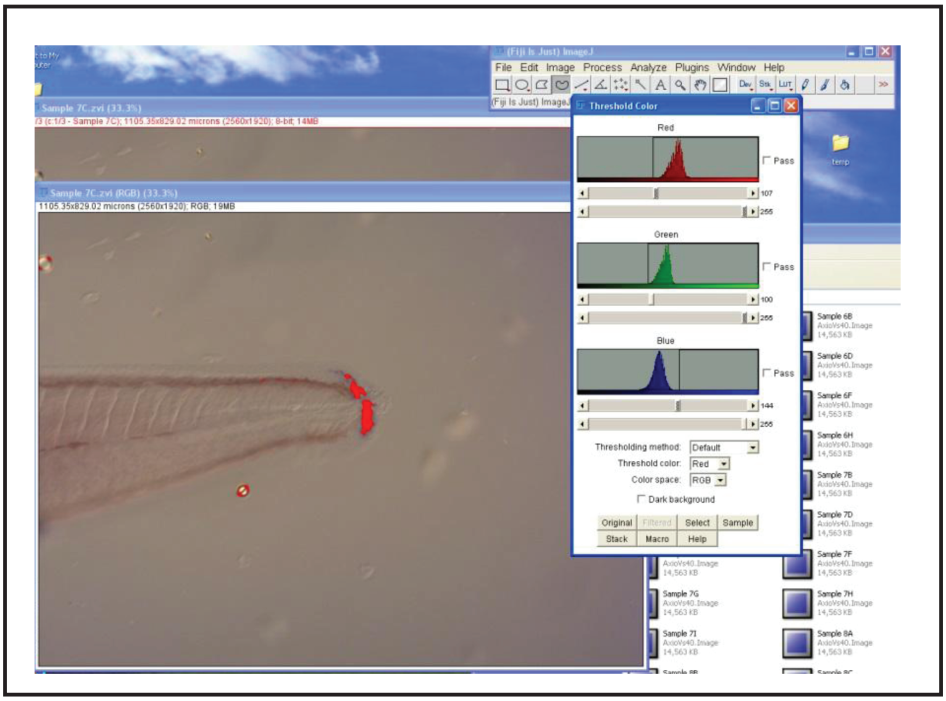

Supplementary Figure 13. Methods image to illustrate RGB-based quantification. The picture shows a screenshot of ImageJ/Fiji to illustrate how RGB threshold limits are set.

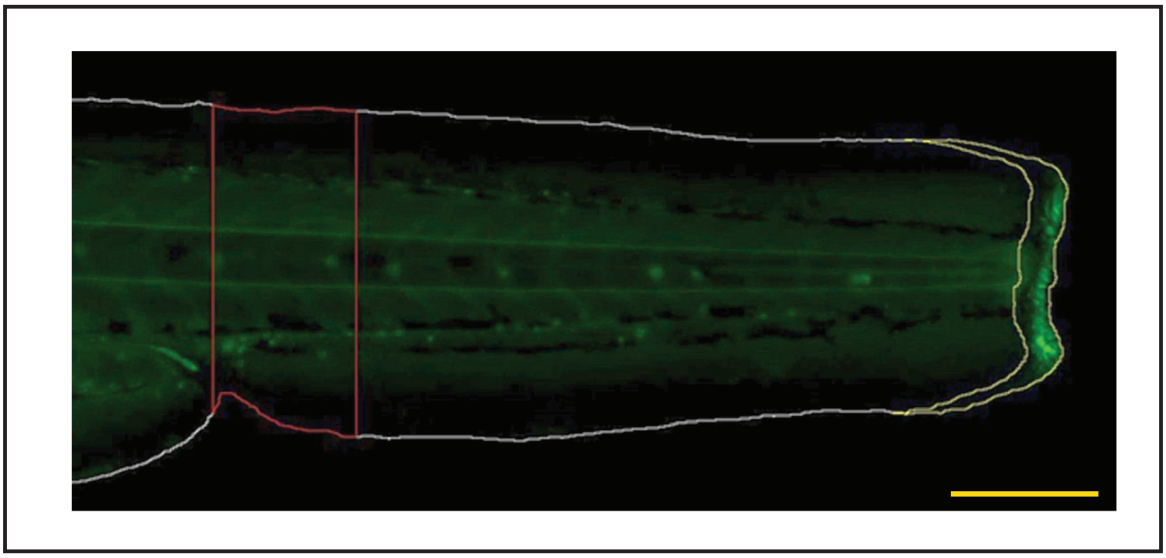

Supplementary Figure 14. Methods image to illustrate fluorescence quantification. The picture shows a screenshot of ImageJ/Fiji Wound-induced H_2O_2 quantification macro to illustrate how regions for quantification are set. The fish, wound area and trunk regions outlined in white, yellow and red, respectively. Scale bar is 200µm.

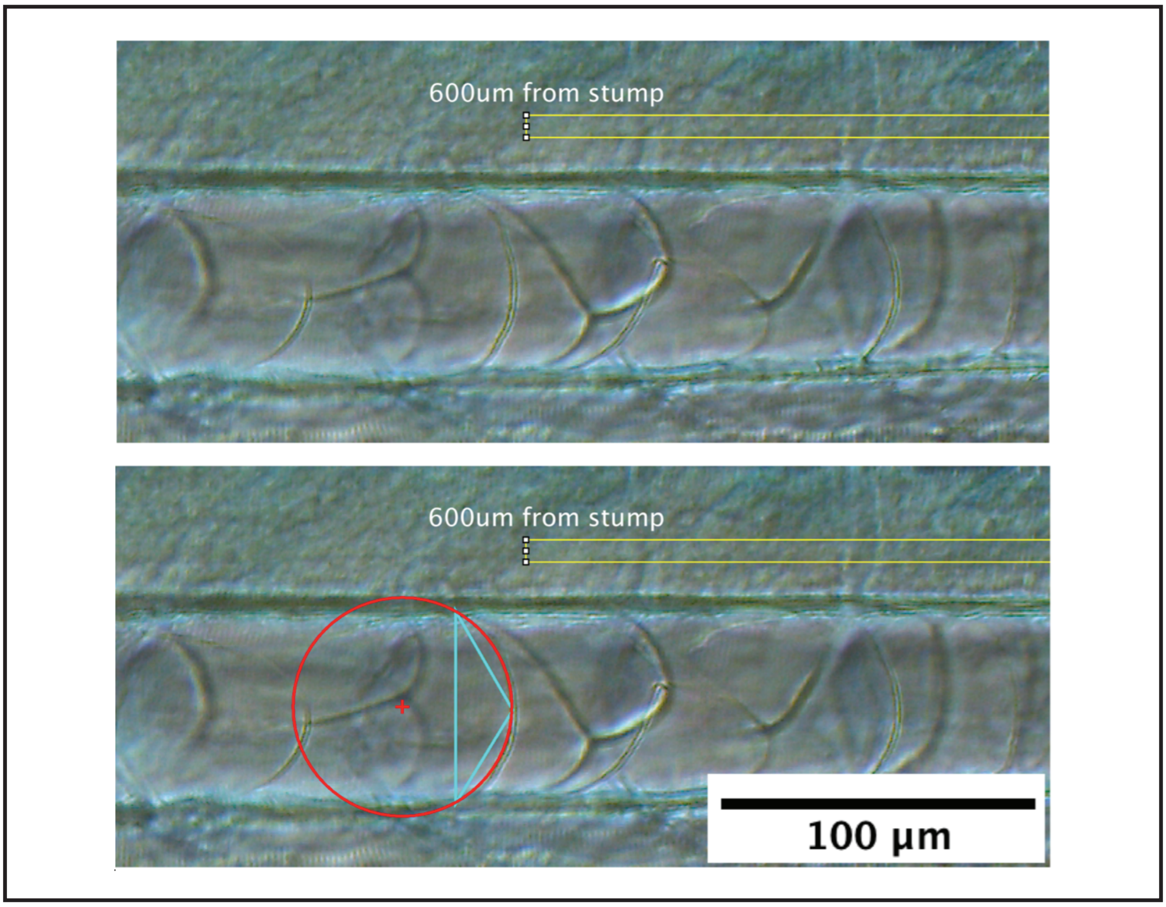

Supplementary Figure 15. Methods image to illustrate Menger curvature measurement. The picture shows screenshots of ImageJ to show how curvature is measured. The example shown was measured as having a curvature of 0.0292μm^{-1}.

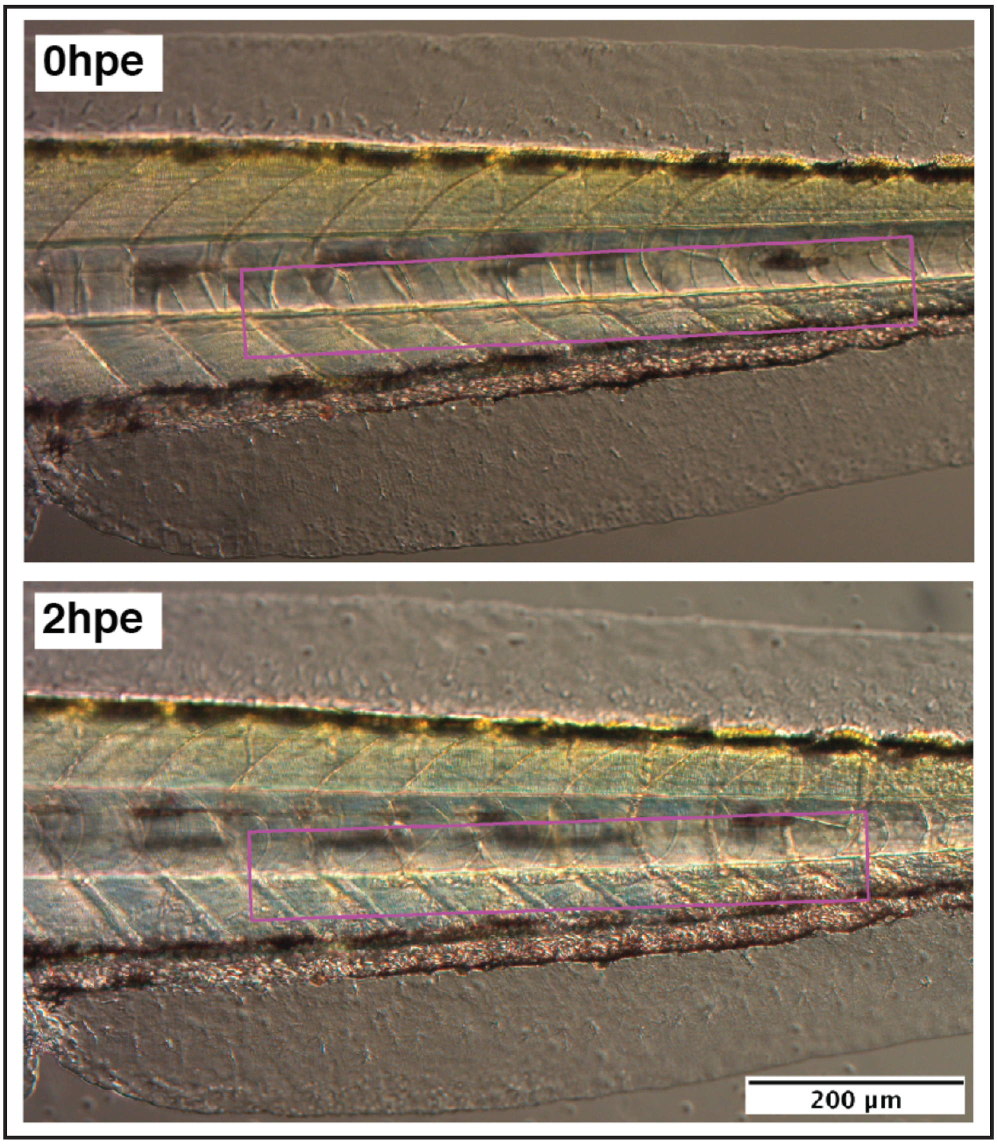

Supplementary Figure 16. Methods image to illustrate measurement of trunk contraction. The picture shows screenshots of ImageJ to show how trunk contraction is measured. The rectangle drawing tool is shown in magenta. This example was measured as 630µm before excision and 580µm after excision, giving a contraction of 8%.

Supplementary Table 1. RNA in situ probe information

gene	sequence
ihhb	GAATTCGAGCTCGGTACCCGGGGATCCGGGGGGTCTNAAATTNGGGGGGTC<br>TNAAATTATCGCTAAATATAGCGACAAAGTCACTAAGTTGGCAACNCNGTTCA<br>ACTGACATCAAATTAATTTCAATTTTTGACATTTTTAATGTGTTTTTGATGTCGT<br>TTGACATCAAGGTGACCTTTTACATCACAAACCCTGAGCTTGTGATCATCTCTT<br>TTGACAACTCTCTCCACCAATCCCTGCGTTTCATTTCAATCCATCATCTCTCAG<br>TTGCCTCTAAGGCCAAAGGGTGGAAGAGTTCTGAGTCTAGCAGTAGTTTTCC<br>CAAACCTATCAGGACCTGAGAGTACCAGTGTAAGCCATTCTTAAGGACCTGAT<br>CTGGTCCGGTCCAGCTATACAGCAAGCGTAGAGGTGCAAAAGCCCAATGAGC<br>AAGTCTTTGCCTGTTCACCGCAGCATAACAAGAGGTGAGCACATCGTTCACC<br>ACTACAGTCCCATGAGCAGTAAGTGGAGGGTATAACCCCTGGTCCTCCCGAA<br>CCCCAACATGAGTGATCTGTGAAAAACGCTTCCTGAGTTTACCCAGCAGCAAA<br>CACTGACCCACTTGAGCATCGCTGGCAAATATCGTCCGGACTGCTCCTGGTT<br>TGGGTTCCCCCCTGTTGGAGCAGTTTCCAACCCTCATAAACAGTAAATGAGC<br>CGCTGTTAAAGACACGCTGGCCCCATCCTCAGTGCGGATCACATAGAAGTGC<br>TTCTGGGTAATGGGGCGACGGTCCAAAAAGGTGAGGACTTCACTATAGATGA<br>GGTCTCCTGTCCCGTCGCTTCCCTCAGAGGCCAGAACCAAGTCTCCTGCCTG<br>CAAATCACGGATTTGTCGATGGGAACCATCCTTCATGGTCACCAGAGCCCTT<br>CCAGGAAAACAGCCTCCTGTTTTTGCTGCTACTGAATGCTCTGATTTGACACT<br>GCAGTGTACGTGGCCTTTGGATTCGTAATAGACCCAGTCGAAGCCTGCTTCC<br>ACCGCAAGACGAGCCAGCATGCGGTACTTGTTGCGATCGCGGTCCGAGGTA<br>GTAATGTCCACCGCTCTGCCCTCATAATGCAGAGATTCTTCAGAGTGTAGTCC<br>GTCCTCATCCCAGCCCTCCGTCACACGCAGACGCACACCGGGCCACAGGTT<br>CATCACCGAGATGGCCAGAGAGTTCAGCTTATCCTTGCAGCGCTGCGTCATC<br>ATGCGGTCCGCACCTGTGTTCTCCTCATCCTTGAAGATGATGTCCGGGTTGT<br>AGTTTGGGGTCAGCTCTTTAAAGCGCTCGGAGCTGGGGGTGACCTTCCCCTC<br>ATATCTCCCGCTCGCCCCCAGCGTTTTCTCGGCTACGTTCGGACTGAACTGC<br>TTATAGGCGAGAGGCGTGAGTTTCCGCGGAGTCCGTCTCTTGCCGTAACCTC<br>TGCCCGGTCCACAGCCGTCGTACGCCGGCGAGAAAGCCAAGATGAAGCCGG<br>TGAGGAGCGCCGCCGCCGTGGAGAGTCTCATTCTAGAGTCGACCTGCAGGC<br>ATGCAAGCTTATTCCCTTTAGTGAGGGTTAATTTATATAGCTTGGCACTGGCC<br>GTCGTTTTAC
shha	GCCACGTTCCCATTTGATACAGGAGCCTGGAGTACCAGTGGACCCCCTCCTG<br>TTGTAAAGTCGCATTGGACCGACTGCTGGAGTTTTGGGGGAACAGGAATGAT<br>GACACGTAATAATAGAGCCTGGCGGGCGCGAAGGCCAAATGCGCAAGCCCC<br>TGGTCCTCTATTACGGCGTAACAGGACGCCAGTATTCTGTCGACCACAATGG<br>TCCCATGTGCAGTCACTGGTGCGAACGAGCCCCGCTGCTCCTCCGTGTATAT<br>CCGCTGCACGATGACAGATTTAAGCTGACCGCTATCATCAACAACCATCACCT<br>TTTGTCCGGCTCTGACACTGCTGGCATACGCGGCGGTCATGGTGTGGAGATC<br>TTCCGTTGAGTTGTCGAGGACAAAAAGGAGGTGAGCGGCGGTGAGGGTGAT<br>CTTTTCAACGGGTTCTTGCGTTTCTATGACGTAAAACACACGTCGCGTCGTGG<br>AGTCTCGGTCTGTGAACATGATGAAGTCGCTGAACACCAGGTTTCCCGCGCT<br>GTCTGCCGCCAGCACCTTGTCTCCGGGGTTCAGGTCCTTCACGGCCTTCTGT<br>CCTCCGTCCTGGAGCGAGACCAGAGCCGAACCTGGGAAACAGCCCCCAGAT<br>TTCGCAGCAACCGAATTTTCTGCTTTGACAGAGCAATGAATGTGGGCTTTGGA<br>CTCGTAATAGACCCAGTCAAATCCAGCCTCCACAGCTAGGCGAGACAGTGTC<br>CCGTATTTGCTCTTGTCTCGGTCAGAGG
ptch1	GGGAGACTGCGGTGAGTTACACGCCATTGGGTCGCTGGAGCTGGGGCTGTG<br>TTCTGGGGGCAGTTCGCCGTTTTTCATAATCTTCTTTAGTTTTGATCTTTTGTT<br>CTGGAACCAGATTTTCACCTGTGTCTGTGTGAGTCCCAGCGATGCGGCGAGC<br>TCGGCTCTTTCTGGAAGCGCGAGGTATTGCGTGTTCTGAAACCTTCTCTGTAA<br>AGCTGCGAGCTGGAAACTGGAGTAAATGGTTCGGGGCTTCCGGACTTTTTTG<br>GGTTTTCCGTTGACCATCCTTACTTCGGGCTCGGCTGTTTCTTTTTCTTGCGG<br>GCTCGGTTGTGATTGCACTCTGTTATATGTTCCTGCGTATTGGTGATACGCTG<br>TGGAGTATGAGCCGTAATCAGGGTAGGATTTTGCAGAGTAATTTCCAGAAGAT<br>CCATTGACTCCGTGGTATTGGTACTGATAGGCATTAAGAGGTTTCCCATAGGT

	GCCCGAGTTCGGTGAACAATAGCCATGATGAACTCCTCCGGCAGGGCTGTAA<br>TAGCCAGAATCCGTGGCTGTGGACTCCGGTAGGGTTGGAGAT
msxc	CATTTTGAATCCTCCGAAAGGACACTGGCAGGACAGGGCGTTATAACAGTTA<br>ATCCAGACGGTGCCGGCCTGCACTGCTGCAGAGATGGTCATGGCCTTGCTG<br>ATATCTCTGGTGAAGACGGCCGCTGCCAGACCGTATTCTGTGTTGTTGGCTC<br>TCTCAATCACTTCTTCAATTGTTTTGAACTTCATGATTTGCTGCACTGGCCCAA<br>ATATCTCCTCCTTGGCGATGCGCATGTGGTCCTTCACATTAGAGAAAACTGTG<br>GGCTCCACGAAGAAGCCTTTAGTAGGAGGAGCTTTGCCTCCACACTCCAGTT<br>TGGCTCCTTCAGTGATGCCACTCTGGATGAGCTCCAGCACACGTCTCTGCTG<br>CTCCTCGCTCACCTGAGGTCCGTGTTCAGTGGTTGGGTCAAAGGGATTTCCC<br>ACTTTCCTCCTCTGTGCTCTCTCAACACTTCTTCTCACAAACTCATCATAAATG<br>GGCTCCTCAACAAAGATACGGGAACCAGCAGTGCAGCATTGACCGTTATTGA<br>AGAAAACCCCCTGATGGGCCTGTTCTAATGCCAGCTCAAAATCAGCATCTGC<br>AAAAATGATGTTGGGACTCTTTCCCCCGAGCTCCAGCGTGACTCTCTTCAGAT<br>TGCTCTTTCCTGCTGCTTCTTGGATCAGCTTGCCTACCTCAGTCGATCCTGTG<br>AACGCCACTTTGTCTATGCCCATGTGCGAAGAGATCGCGGCTCCTGCGGTTG<br>GCCCATAGCCTGGCAAAATATTGACGACTCCCGGTGGAAACCCAGCCTCTTT<br>GATCAGAGCGCCGAGGTAGAGGCAGGTGAGAGGGGTTTGCTCAGCAGGTTT<br>CAGGACAACCGTGTTCCCGCAGCTCAGCGCCGGCCCCAATTTCCATGCTGTC<br>ATCACCAGGGGGAAGTTCCAAGGAATGAT
raldh2	CATTTTGAATCCTCCGAAAGGACACTGGCAGGACAGGGCGTTATAACAGTTA<br>ATCCAGACGGTGCCGGCCTGCACTGCTGCAGAGATGGTCATGGCCTTGCTG<br>ATATCTCTGGTGAAGACGGCCGCTGCCAGACCGTATTCTGTGTTGTTGGCTC<br>TCTCAATCACTTCTTCAATTGTTTTGAACTTCATGATTTGCTGCACTGGCCCAA<br>ATATCTCCTCCTTGGCGATGCGCATGTGGTCCTTCACATTAGAGAAAACTGTG<br>GGCTCCACGAAGAAGCCTTTAGTAGGAGGAGCTTTGCCTCCACACTCCAGTT<br>TGGCTCCTTCAGTGATGCCACTCTGGATGAGCTCCAGCACACGTCTCTGCTG<br>CTCCTCGCTCACCTGAGGTCCGTGTTCAGTGGTTGGGTCAAAGGGATTTCCC<br>ACTTTCCTCCTCTGTGCTCTCTCAACACTTCTTCTCACAAACTCATCATAAATG<br>GGCTCCTCAACAAAGATACGGGAACCAGCAGTGCAGCATTGACCGTTATTGA<br>AGAAAACCCCCTGATGGGCCTGTTCTAATGCCAGCTCAAAATCAGCATCTGC<br>AAAAATGATGTTGGGACTCTTTCCCCCGAGCTCCAGCGTGACTCTCTTCAGAT<br>TGCTCTTTCCTGCTGCTTCTTGGATCAGCTTGCCTACCTCAGTCGATCCTGTG<br>AACGCCACTTTGTCTATGCCCATGTGCGAAGAGATCGCGGCTCCTGCGGTTG<br>GCCCATAGCCTGGCAAAATATTGACGACTCCCGGTGGAAACCCAGCCTCTTT<br>GATCAGAGCGCCGAGGTAGAGGCAGGTGAGAGGGGTTTGCTCAGCAGGTTT<br>CAGGACAACCGTGTTCCCGCAGCTCAGCGCCGGCCCCAATTTCCATGCTGTC<br>ATCACCAGGGGGAAGTTCCAAGGAATGAT
axin2	ATCCGCACACCAGCTTTAGACCTCCTAGACCATTGGGATTCACATGACTGGG<br>GTTCTCGCACCCAGTTCTGACATACTCGAGGTATATGTCCGAGGTCAAAAACA<br>TCTGATAGGCATTCTCCTCCATAGCCGTCTGGATCTCCATTTGAGCCTGATCA<br>AACATTGCAGAGTCAATCTGCTGACGCTTAATATTATCCCTAATAAAGGTCTTA<br>GTGGCAGGTTTAAGCTGCTTGGCCACAATGCTGTTGTTCTCAATATACCGCTT<br>GTAAATGGCTTTGGCAACTCTGTGCGTTTTGGTATCCTTGAGGTCCATTTGTC<br>TGAAACCATTGCAGGCAAACCAAAAATCTAAAGTGTCCACACATTTCTCGCGT<br>TCAAGGTATGCCCGGAAAAGTTGAGCACCATCCTGGTCCCCGAGAAGAAAAT<br>GCAAAGATTTGGTCCACCGGGCAAGAGGGGAGTCCGGGGATGCGCTGCCCT<br>CAGGTTCCCCAAGTCCATCCTCATCCCTCCTGGCCGTGGAACAACGAAGATC<br>AGCCATAATGGTCTTGACAGGATCCTTCGGTCTCATCATGGCCAGTTTGCTG<br>GGATGGTGGCATGTCGTCTCACCCTCTTCC
pea3	CTGCAGGTGCTGCTGCCTCTGTCCGTCTTCCTTCCCCTCTCAGTCTGCGTGG<br>AGCTCATTAAAACACAGCCTAAATGACCACAAACGGTTAAGGCATGGATTATA<br>AGATGGATGGATATCTGGACCAGCAAGTGCCTTATACTTTAGCTAATAGGTCG<br>CAAGGAAATGGGCCCCTAAATAGACTGTTGATGGCGACAAAAAGGAAATACA<br>TGGACGCAGAATTACCCCCTCAGGAATCTGAAGACCTCTTTCAGGATTTAAGC<br>CAACTTCAGGAGACCTGGCTCACCGAAGCTCAAGTTCCTGACAGCGATGAGC<br>AGTTTGTTCCTGACTTTCACTCAGAGAACTCAGTGGCATTCCACAGCCCGCCT<br>GTGAAGATTAAGAAAGAGCCGCAGAGTCCCGGATCAGACCCCAGCCAGTCCT<br>GCAGCCACAAGCAAAGCTTCAGCTACCCCAATGGAGAGCAGTGCCTTTACGC<br>CAGTGCCTATGAGCAGAAGAGAGCAGCCGTGGCAGGAGCAGGAGGATCTAA

	GAGCTCATGTCCAGGGACGCCCATGTCCCCTATGCAGCATTATTCCCCCAAA<br>CCAACAGTGGGAACTCGGCAGGAATCAGGGTATATGAATCCACCCTCAGCCA<br>GCCAATCCCACGCCTGCCACAGCCACAGTTACCCCATGAACCCCAGTTCCAG<br>GTTCCCTTCCGGCTCGGCAGAGATGTGCCCACCATTCGCTTCCCAAGGCCAG<br>GCCCTGCAGCGCATGCATCCTGCCCATGCCAGCGGAGGCGGTTACCATCGG<br>CAACACTCTGACCCCTGCTTGCCTTATCCTCCTCAGCAGACCTTTAAGCAAGA<br>GTACATGGACCCTCTGTATGACCGAGCGGCCCACATTAACGGACCACAGCCT<br>CAGAGGTTTCCTCCTGCCCATATGATGGTCAAACAGGAGCCCACCGACTACA<br>CTTACGAACCTGATGTGCCTGGCTGCCCATCCATGTACCATCACAACGAAGG<br>CTACTCCAACCCACAGCACAACAGTGAAGGCTACATGTTTGAAAATGATTCCC<br>GTGTTGTGCCAGAGAAATTTGAAGGTGAGGTGAAACAAGAAGGGGGCAGTGT<br>GTTTCGTGAAGGTGCCCCCTATCAGCGTCGTGGCTCACTTCAACTATGGCAG<br>TTCTTGGTTGCCCTTCTTGATGACCCCAGCAATGCCCACTTCATCGCATGGAC<br>GGGCCGTGGCATGGAATTCAAACTCATTGAGCCAGAGGAGGTGGCAAGACT<br>CTGGGGGATGCAGAAGAACCGTCCAGCCATGAACTATGACAAACTGAGTCGC<br>TCTTTGCGTTACTATTATGAGAAGGGAATTATGCAAAAGGTGGCTGGTGAGCG<br>TTATGTTTACAAATTTGTGTGTGAGCCAGAAGCTTTGATAACCTTGGCTTTTCC<br>CGACAATCAGCGGCCAAGTCTGAAGGCGGAATTTGAGCGCTACGTCAACGA<br>GGAGGACACTGTGCCACTGTCCCACCTCGATGAGGGAGTTTCTTACCCTCCC<br>GAACCAGCTGCCACCAACATGGGCCCTCAGCCCTACTCCAAAGGCTACATGT<br>ACTAATGCCACCGACATTCTCCTTTGTTTCCCATCCCAGTTTACCACCCGCTG<br>CACCTATTGCACATGCCCACCCCATCCTCTTGTATATAAGGCTATGTTTTATGT<br>TTTTGTTTTGATTAAATTAATCTCATTAGGGACTCTGCATTCCTCTCACTTCTGA<br>GGCCTATCTAATCTAAACACAATACTGTGGTTCAAGAGATTGCTTAATAAACC<br>CAATATTACTACTATAAACGAGGGGGCCCGTA
tcf7	ACCAGTCCGTCTGTTGGTTCAGGCCGAAGCGAGCGCGGCATTTCTTTGGAGA<br>GCCAGGCCCTGTACTGGAGTCCTGCTGCTTGTCCCGTTTCCTCCGCTTCTTTT<br>TGCCCAAAGCACTCACGTAGTTGTCACGGGCCGACCAGCTGGGGTAGAGCT<br>GCACGTGCAGCTGCCGCTCCTTACGGGCCAGCTCATAGTACTTGGCCTGTTC<br>CTCACGGGTCAGAGCGTGCCACCGGCGGCCCAGGATCTGGTTGATGGCGGC<br>GCTCTCCTTCAGTGTGCACTCTGCGATCACTTTAGCGCGCATCTCCTTCATGT<br>ACAGCATGAACGCATTCAGAGGCTTCTTGATCACCGGCTTCTTGGGCTCCTT<br>CTCCCGCTTGGCCTCCGCATGAGACTTGTTATAAATGCTCCTGTCGAACTGAT<br>CGTGCTCCTGCTTTCCTGAAGGGGGCACTATAGCGGGGTGAGGGATTCCTGT<br>GGGGTGCATACCGGACTGCAGCATCAGAGAGTGAGAGAACCAGTTCATGGA<br>GGGCGTGATCTGACCCTGAGGCAGCGAGTAGAAACCCGAAATCTCCTGCGT<br>CTGATGTCTGTGAACACCTGGTTTCTGTCCTCCGTCTGTGGGCATGTGTGTG<br>GGACTGGGGTTGAAGTGTTCATACGGCAGCAGCGGCGTCAGCGGATGCATC<br>CCCTGCTGGACCACCGACACCTTGTTGGAAGGTGGAGAGACGGGTCCGTTG<br>GGCAGATACGGCTCCGGCAGCATCAGGAACGGGTATCCGGAGTACGGCGCT<br>TTATACATCCCTCCATCGTGATGTTTGGGCACGTCGTCCAGATGTTCTCGTTT<br>ATCGCTGTAGATCCTCTGCTCGTCCTGCTGTCCTCTTCTGATCACGGCTGGG<br>CTCTGGCTGATCTCGGTCTCGCTGACCAGTGATGA
myod	CAAAGAAACCCTCCGAGGTCCTGCGAATTTTAGACTGTAGTTTTGAAACGTAC<br>GTTATTTCGGGATCTGAAGGACTTTGTCTTACTATTCCTAATTTTGGTTTTTATT<br>TTTTTTACAAATTTACTACTTTGGAAACATTCAAGTACATTTCTTGCTTTTTAAA<br>CTTTAACACAAAAAAGATGGAGTTGTCGGATATCCCCTTCCCCATCCCATCAG<br>CTGATGACTTCTACGACGACCCTTGCTTCAACACCAACGACATGCACTTCTTT<br>GAAGACTTGGACCCCAGGCTTGTTCACGTGAGCCTGCTCAAACCCGACGAGC<br>ATCACCACATCGAGGACGAGCACGTGAGGGCGCCCAGTGGGCATCATCAGG<br>CCGGCAGGTGCCTACTGTGGGCATGCAAAGCTTGCAAGAGAAAAACTACCAA<br>TGCTGACCGTCGCAAAGCCGCCACCATGAGGGAGAGGAGGCGACTGAGCAA<br>GGTCAACGACGCTTTCGAGACCCTCAAGAGATGCACGTCCACCAACCCGAAC<br>CAGAGGCTGCCCAAAGTGGAGATTCTGAGAAACGCCATTAGTTATATCGAGT<br>CTCTGCAGGCTCTTCTCAGAAGTCAAGAGGATAACTACTATCCCGTTCTGGAA<br>CATTACAGTGGAGACTCTGATGCTTCCAGTCCGAGATCCAACTGCTCTGATG<br>GCATGATGGATTTTATGGGCCCAACGTGTCAGACGAGAAGACGGAACAGCTA<br>TGACAGCTCTTACTTCAATGACACACCAAATGCTGACGCACGGAATAATAAAA<br>ACTCAGTGGTGTCGAGTTTGGATTGTCTGTCCAGCATCGTGGAGCGAATTTC<br>CACAGAGACTCCTGCATGTCCCGTGCTGTCAGTACCGGAGGGGCACGAAGA

	GAGCCCGTGTTCTCCGCATGAGGGATCTGTCCTGAGTGACACCGGAACCAC<br>CGCACCGTCCCCGACCAGCTGCCCTCAACAGCAGGCTCAGGAAACCATTTAT<br>CAAGTGCTTTAAAATTCTGCAACATTTCAAAACAAATTGAAAAAGACAATCTGA<br>ATGAAGAAACTTTCAGCAGAAAAAGGCGAATCCGACCTTTAACGACAAAAGAA<br>AGACTTTTGATCCACTGCTGGAAACTAGGAAAGAATGCTTTCTTTCTTTCTTTC<br>TTTCTTTCTTTCTTTCTTTCTTTCTTTCTTTCTTTCTTTCTTTCTTTCTTTCTTTCT<br>TTCTTTCTTTCATAGACTAATTCTTTTTTTCTTTCTTTCTTTCTTTCTTTCTTTCTT<br>TCTTTCTTTCTTTTTTTCTTTCTTTCTTTCTTTCTTTCTTTCTTTCTTTCTTTCTTT<br>CTTTCTTTCTTTCTTTCTTTATGCGGTGGAAAATGTAATTCCAATATGAGAAAA<br>GACTCAGCAGATCAGCAGAAGTGAGTTTTTTAACTAAGCTTATTTATATTGTTA<br>TATCCACGTGAAAATGTGACCATATTTTTTTCCCTTTGTGAATATATTTTCTGTC<br>ACCATCACCTTTTATTTTCCTAATTATTTACGGAAAAAATCGTAATCCAGATAC<br>GAATAGTAGGACCATTTTTGTATATGTGTAAATAAGATCTGTTTTGTTAAAGCG<br>ACGAAAACGAACAAACATATTATTGTATTTAATGATGCCTGTTTGAAACACTAG<br>TTTGTTGTGTCTTGTGTTAAACTTTATATTTATACTTCTTAAACGAGTGAATGAC<br>GGATAAATAAAAACAACTATTTATACTGGAAAA
wnt10a	CTTTGACCCCATCCTCAATGCCAACACGGTGTGTTTGACCCTGCCAGGCCTG<br>ACAAAGAAACAGCTGGACGTTTGTATGAGAAATCCAGATGTGACGGCCTCAG<br>CCATACAGGGCATTCAGATTGCCATTCATGAGTGCCAGCATCAGTTCCGAGG<br>GCACCGCTGGAACTGCTCAAGTTTGGAGACCAGGAATAAAATCCCTTACGAG<br>AGTGTGGTCTTCAGCAGGGGTTTCAGAGAAAGCGCATTTGCTTATGCCATTG<br>CTGCAGCTGGTGTGGTGCATGCCGTGTCTAATGCCTGTGCCATGGGCAAACT<br>GAAGGCCTGCGGCTGTGACGAGAAGCGCAGAGGAGACGAGGAGGCGTTCA<br>GAATCAAGCTCAACCGTCTGCAGCTGGAGGCCATCAATCGAGGCAAAGGCAT<br>GGTGCACGGTGTGATGGAGCACTTCCCTGCCGAGGCACTCGGCCCCCAGGA<br>CTCCTGGGAGTGGGGCGGCTGCAGTCCCAACGTGGAGTATGGGGAGCGCTT<br>CTCCAAGGACTTCCTGGACTCCCGCGAGACGTACAGAGACATTCACTCCAGG<br>ATGAGACTTCATAACAACAGAGTTGGCAGGCAGGTTGTGGTGGACCACATGA<br>GGAGGAAATGCAAATGCCATGGTACATCGGGCAGCTGTCAGCTGAAGACCTG<br>TTGGCAGGTGACTCCAGAGTTCAGAACGGTGGGCTCGCTGCTGAAGGAGCG<br>CTTCAACGTGGCCACGCTAATCAAAGCCCACAACAGAAACACAGGTCAGGTG<br>GAGAATGCCCACCACACACACCGGCGGAGAGCCAACATCAATGACCTGGTCT<br>ACTTCGAGAAGTCCCCTGACTTCTGTGAGAGGGACCTTGGGTCGGACTCTGC<br>CGGGACGCAAGGCCGGATCTGTAACA
fgf10a	GTTCCTGTGTTCGGCTCTGCCTGTGGCCTGCCATGACACCCACAGGGCCATC<br>CGTGCCCCGAGGGGCACCAACTCCTCATCGTCTGCCGTGGTGGGGCGGCAT<br>GTGCGCAGCTACAACCACCTCACGGGGGACGTGCGCAGGAGGAAACTCTTC<br>TCCTACCAGAAGTTCTTTCTCAGGATCGATAAGAACGGAAAAGTCAATGGCAC<br>CAAAAGCAAGGACGATCCGTACAGTACACTCGAAATCAAGTCTGTGGATGTG<br>GGCATCGTTGCCATCAAGGGGATTCAAAGCAATTACTACCTTGCAATTAACAA<br>GAAAGGGGTGGTCTACGGGGCGAGGGATTTCGGCATTGACTGCAAGCTGAT<br>AGAGAGGATAGAGGAGAACAGGTACAACACCTATGCCTCGGCAGAATGGATG<br>AACAAGAAGAAGCACATGTTCGTAGGTCTGAGCGCCAACGGGAGGCCGATG<br>AGGGCCAAAAAGACCCGGAGAAAAAACACAGCCACACACTTTCTCCC
