# Supplementary material for: Damage-induced reactive oxygen species enable zebrafish tail regeneration by repositioning of Hedgehog expressing cells
Source: Nat Commun. 2018 Oct 1;9:4010. doi: 10.1038/s41467-018-06460-2 (PMC6167316; doi:10.1038/s41467-018-06460-2)
Supplement: Supplementary file 3 — Description of Additional Supplementary Files [file 41467_2018_6460_MOESM3_ESM.docx]

**Description of Additional Supplementary Files**

File Name: Supplementary Movie 1

Description: Tissue movement following tail excision. This movie shows the rapid repositioning of notochord cells to form the notochord bead. The white arrow marks one cell's movement (1.5μm/minute). Note that the notochord cell membranes are bowed towards the anterior at the start of the movie and rapidly change to bow posteriorly within the first five minutes. This may indicate that pressure is building up within the notochord, and that subsequent notochord cell movement is passive rather than an active migration. Green dots mark reference points within the fin fold epithelia and show the movement of the tissue towards the posterior. Over the duration of the movie the path between these dots shrinks by 11%. Red dots mark individual fin mesenchymal cells, these move together by 9% during the movie. Measurement of the length of the dorsal edge of the body using melanocyte position also shows a contraction of 9%. Together these indicate that the trunk shrinks by approximately 10% along the anterior/posterior axis during the first three hours after excision. Measurements along the dorsal/ventral axis of each somite show that the trunk becomes wider by approximately 7%. Consistent with this, the angle of the somites increases by 5% on average. The larvae was mounted in 1% low melt agarose at 33°C, then when hardened the agarose was cleared away so that only the head remained embedded. The tail was excised and the fish was covered with a coverslip. Mounting took 4 minutes, during which time the initial wound closure is likely to have taken place. Images were captured every 30 seconds for 170 minutes at 28.5°C. Replay is set to 20 frames per second. Anterior is to the left.

File Name: Supplementary Software 1

Description: ImageJ macro which is used to blind images to the researcher before quantification takes place.

File Name: Supplementary Software 2

Description: ImageJ macro which is used to quantify wound-induced H_2_O_2_.

File Name: Supplementary Software 3

Description: ImageJ macro which is used set the RGB values for RNA in situ quantification.

File Name: Supplementary Software 4

Description: ImageJ macro which is used to quantify expression from RNA in situ images.

File Name: Supplementary Software 5

Description: ImageJ macro which is used to measure the curvature of cell membranes.
