# Supplementary material for: Damage-induced reactive oxygen species enable zebrafish tail regeneration by repositioning of Hedgehog expressing cells
Source: Nat Commun. 2018 Oct 1;9:4010. doi: 10.1038/s41467-018-06460-2 (PMC6167316; doi:10.1038/s41467-018-06460-2)
Supplement: Supplementary file 5 — Supplementary Software 1 [file 41467_2018_6460_MOESM5_ESM.docx]

Supplementary Software 1

Renaming images for blind analysis

setBatchMode(true);

function isImage(filename) {

extensions = newArray("tif", "tiff", "jpg", "bmp", "czi", "zvi");

result = false;

for (i=0; i<extensions.length; i++) {

if (endsWith(toLowerCase(filename), "." + extensions[i]))

result = true;

}

return result;

}

path=getDirectory("Choose a Directory");

list=getFileList(path);

outputpath=path+"BlindedÄÄ";

File.makeDirectory(outputpath);

print("Original File,Blinded Name");

names=newArray();

for(i=0;i<list.length;i++){

if (isImage(path+list[i])) {

open(path+list[i]);

//Image analysis

rawpic=getTitle();

temp=floor(random*1000000);

for (j=0;j<names.length;j++) {

if (temp==names[j]) {

temp=temp+random;

}

}

names=Array.concat(names,temp);

rename(temp);

saveAs("Tiff",outputpath+temp+".tif");

close(temp+".tif");

print(rawpic+","+temp);

//Image analysis

}

}

selectWindow("Log");

save(outputpath+"/Blinding Key.txt");

File.rename(outputpath+"/Blinding Key.txt", outputpath+"/Blinding Key.csv");

selectWindow("Log");

run("Close");

setBatchMode(false);

exit("Macro Completed Successfully");
