# Supplementary material for: Damage-induced reactive oxygen species enable zebrafish tail regeneration by repositioning of Hedgehog expressing cells
Source: Nat Commun. 2018 Oct 1;9:4010. doi: 10.1038/s41467-018-06460-2 (PMC6167316; doi:10.1038/s41467-018-06460-2)
Supplement: Supplementary file 6 — Supplementary Software 2 [file 41467_2018_6460_MOESM6_ESM.docx]

Supplementary Software 2

Quantification of wound-induced H₂O₂

setBatchMode(true);

function isImage(filename) {

extensions = newArray("tif", "tiff", "jpg", "bmp", "czi", "zvi");

result = false;

for (i=0; i<extensions.length; i++) {

if (endsWith(toLowerCase(filename), "." + extensions[i]))

result = true;

}

return result;

}

IJ.deleteRows(0, nResults);

setForegroundColor(255, 255, 255);

run("Set Measurements...", "area mean standard modal min shape integrated median limit

display redirect=None decimal=3");

path=getDirectory("Choose a Directory");

ROIpath=path+"Fixed Wound ROI\\";

File.makeDirectory(ROIpath);

list=getFileList(path);

for(i=0;i<list.length;i++){

if (isImage(path+list[i])) {

open(path+list[i]);

////Scaling

//for 30X zoomscope:

run("Set Scale...", "distance=701 known=1000 pixel=1 unit=micron");

///////////////////////////////////////////

////Image Analysis

//for pics with wound left, trunk right

woundlength = 50; //in microns

toUnscaled (woundlength);

rawpic = getTitle();

run("Split Channels");

close("C1-"+rawpic);

selectWindow("C2-"+rawpic);

rename(rawpic);

selectWindow(rawpic);

run("Duplicate...", "title=mask");

run("Auto Threshold", "method=Triangle white");

run("Make Binary");

run("Close-");

run("Open");

run("Fill Holes");

run("Erode");

run("Erode");

run("Erode");

run("Erode");

width = getWidth;

height = getHeight;

getPixelSize(unit, pw, ph);

ParticleMin=(50000);

ParticleMax=(3000000);

run("Analyze Particles...", "size="+ParticleMin+"-"+ParticleMax+" add");

FishCount=roiManager("count");

if (FishCount==0) {

setTool("wand");

setBatchMode("exit & display");

run("Select None");

roiManager("Show None");

waitForUser("Macro Paused", "Select the fish ROI, then click OK");

while (selectionType()==-1) {

setTool("wand");

waitForUser("Macro Paused", "Select the fish ROI, then click OK");

}

roiManager("Add");

setBatchMode(true);

}

else if (FishCount>1) {

setBatchMode("exit & display");

run("Select None");

roiManager("Deselect");

roiManager("Show None");

setTool("wand");

waitForUser("Macro Paused", "Select the fish ROI, then click OK");

while (selectionType()==-1) {

run("Select None");

roiManager("Deselect");

roiManager("Show None");

setTool("wand");

waitForUser("Macro Paused", "Select the fish ROI, then click OK");

}

roiManager("Add");

setBatchMode(true);

}

roiManager("select", (roiManager("count")-1));

roiManager("rename", "Fish");

fish = roiManager("index");

Roi.getBounds(fx,fy,fw,fh);

roiManager("select", fish);

run("Make Inverse");

Roi.setName("Background");

roiManager("Add");

roiManager("select", (roiManager("count")-1));

BG = roiManager("index");

makeRectangle(fx+1000/pw, 0, 200/ph, height);

Roi.setName("TrunkCol");

roiManager("Add");

roiManager("select", (roiManager("count")-1));

trunkcol = roiManager("index");

roiManager("select", newArray(fish, trunkcol));

roiManager("AND");

roiManager("Add");

roiManager("select", (roiManager("count")-1));

roiManager("rename", "Trunk");

trunk = roiManager("index");

roiManager("select", fish);

roiManager("Add");

roiManager("select", (roiManager("count")-1));

roiManager("rename", "Shifted");

shifted = roiManager("index");

setSelectionLocation(fx+woundlength, fy);

roiManager("Update");

roiManager("select", newArray(fish,shifted));

roiManager("AND");

roiManager("Add");

roiManager("select", (roiManager("count")-1));

roiManager("rename", "Exclude");

exclude = roiManager("index");

roiManager("select", newArray(fish,exclude));

roiManager("XOR");

roiManager("Add");

roiManager("select", (roiManager("count")-1));

roiManager("rename", "PotentialWound");

potentialwound = roiManager("index");

/////////////////////////////For auto setting of wound limit

makeRectangle(fx, fy, 200/pw, fh);

Roi.setName("Gap");

roiManager("Add");

roiManager("select", (roiManager("count")-1));

gap = roiManager("index");

Roi.getBounds(gx,gy,gw,gh);

/////////////////////////////For auto setting of wound limit

makeRectangle(gx, 0, gw+woundlength, height);

Roi.setName("WoundCol");

roiManager("Add");

roiManager("select", (roiManager("count")-1));

woundcol = roiManager("index");

roiManager("select", newArray(potentialwound,woundcol));

roiManager("AND");

roiManager("Add");

roiManager("select", (roiManager("count")-1));

roiManager("rename", "Wound");

wound = roiManager("index");

Roi.getBounds(wx,wy,ww,wh);

selectWindow(rawpic);

roiManager("select", wound);

roiManager("measure");

roiManager("select", trunk);

roiManager("measure");

roiManager("select", BG);

roiManager("measure");

roiManager("Deselect");

roiManager("Delete");

close(rawpic);

close("mask");

}

////Image Analysis

///////////////////////////////////////////

}

setBatchMode(false);

getDateAndTime(year, month, dayOfWeek, dayOfMonth, hour, minute, second, msec);

saveAs("results", path + "Fixed Wound ROI Results - "+dayOfMonth + "-" + month+1 + "-" +

year +".csv");

selectWindow("Results");

run("Close");

exit("Macro Completed Successfully");
