# Supplementary material for: Damage-induced reactive oxygen species enable zebrafish tail regeneration by repositioning of Hedgehog expressing cells
Source: Nat Commun. 2018 Oct 1;9:4010. doi: 10.1038/s41467-018-06460-2 (PMC6167316; doi:10.1038/s41467-018-06460-2)
Supplement: Supplementary file 7 — Supplementary Software 3 [file 41467_2018_6460_MOESM7_ESM.docx]

Supplementary Software 3

Setting the RGB threshold limits

Dialog.create("Select RGB Filter Parameters");

items = newArray("Pass", "Stop");

Dialog.addRadioButtonGroup("Red:", items, 1, 2, "Pass");

Dialog.addNumber("Min", 0);

Dialog.addNumber("Max", 255);

Dialog.addMessage("\n");

Dialog.addRadioButtonGroup("Green:", items, 1, 2, "Pass");

Dialog.addNumber("Min", 0);

Dialog.addNumber("Max", 255);

Dialog.addMessage("\n");

Dialog.addRadioButtonGroup("Blue:", items, 1, 2, "Pass");

Dialog.addNumber("Min", 0);

Dialog.addNumber("Max", 255);

Dialog.show;

filter=newArray(3);

low=newArray(3);

high=newArray(3);

filter[0]=Dialog.getRadioButton();

filter[1]=Dialog.getRadioButton();

filter[2]=Dialog.getRadioButton();

low[0]=Dialog.getNumber();

high[0]=Dialog.getNumber();

low[1]=Dialog.getNumber();

high[1]=Dialog.getNumber();

low[2]=Dialog.getNumber();

high[2]=Dialog.getNumber();

print("\\Clear");

print(filter[0]);

selectWindow("Log");

saveAs("text",getDirectory("macros")+"/Temp/RGBfilter0.txt");

print("\\Clear");

print(filter[1]);

selectWindow("Log");

saveAs("text",getDirectory("macros")+"/Temp/RGBfilter1.txt");

print("\\Clear");

print(filter[2]);

selectWindow("Log");

saveAs("text",getDirectory("macros")+"/Temp/RGBfilter2.txt");

print("\\Clear");

print(low[0]);

selectWindow("Log");

saveAs("text",getDirectory("macros")+"/Temp/RGBlow0.txt");

print("\\Clear");

print(low[1]);

selectWindow("Log");

saveAs("text",getDirectory("macros")+"/Temp/RGBlow1.txt");

print("\\Clear");

print(low[2]);

selectWindow("Log");

saveAs("text",getDirectory("macros")+"/Temp/RGBlow2.txt");

print("\\Clear");

print(high[0]);

selectWindow("Log");

saveAs("text",getDirectory("macros")+"/Temp/RGBhigh0.txt");

print("\\Clear");

print(high[1]);

selectWindow("Log");

saveAs("text",getDirectory("macros")+"/Temp/RGBhigh1.txt");

print("\\Clear");

print(high[2]);

selectWindow("Log");

saveAs("text",getDirectory("macros")+"/Temp/RGBhigh2.txt");

print("\\Clear");

selectWindow("Log");

run("Close");

showMessage("in situ macro","RGB filters successfully set");
