# Supplementary material for: Damage-induced reactive oxygen species enable zebrafish tail regeneration by repositioning of Hedgehog expressing cells
Source: Nat Commun. 2018 Oct 1;9:4010. doi: 10.1038/s41467-018-06460-2 (PMC6167316; doi:10.1038/s41467-018-06460-2)
Supplement: Supplementary file 8 — Supplementary Software 4 [file 41467_2018_6460_MOESM8_ESM.docx]

Supplementary Software 4

Quantification of images from pre-set RGB threshold limits

setBatchMode(true);

function isImage(filename) {

extensions = newArray("tif", "tiff", "jpg", "bmp", "czi", "zvi");

result = false;

for (i=0; i<extensions.length; i++) {

if (endsWith(toLowerCase(filename), "." + extensions[i]))

result = true;

}

return result;

}

path=getDirectory("Choose a Directory");

list=getFileList(path);

//load RGB filter

filter=newArray(3);

low=newArray(3);

high=newArray(3);

f0=File.openAsString(getDirectory("macros")+"/Temp/RGBfilter0.txt");

f1=File.openAsString(getDirectory("macros")+"/Temp/RGBfilter1.txt");

f2=File.openAsString(getDirectory("macros")+"/Temp/RGBfilter2.txt");

l0=File.openAsString(getDirectory("macros")+"/Temp/RGBlow0.txt");

l0=parseFloat(l0);

l1=File.openAsString(getDirectory("macros")+"/Temp/RGBlow1.txt");

l1=parseFloat(l1);

l2=File.openAsString(getDirectory("macros")+"/Temp/RGBlow2.txt");

l2=parseFloat(l2);

h0=File.openAsString(getDirectory("macros")+"/Temp/RGBhigh0.txt");

h0=parseFloat(h0);

h1=File.openAsString(getDirectory("macros")+"/Temp/RGBhigh1.txt");

h1=parseFloat(h1);

h2=File.openAsString(getDirectory("macros")+"/Temp/RGBhigh2.txt");

h2=parseFloat(h2);

filter[0]=f0;

filter[1]=f1;

filter[2]=f2;

low[0]=l0;

low[1]=l1;

low[2]=l2;

high[0]=h0;

high[1]=h1;

high[2]=h2;

//load RGB filter

print("Channel,Low,High,Filter");

print("Red,"+low[0]+","+high[0]+","+filter[0]);

print("Green,"+low[1]+","+high[1]+","+filter[1]);

print("Blue,"+low[2]+","+high[2]+","+filter[2]);

print("\n");

print("Image,Area (px2),Mean Red,Mean Green,Mean Blue");

for(i=0;i<list.length;i++){

if (isImage(path+list[i])) {

open(path+list[i]);

//Image analysis

run("Set Scale...", "distance=0 known=0 pixel=1 unit=pixel");

rawpic=getTitle();

run("RGB Color");

RGBpic=getTitle();

run("Duplicate...", "title=Original");

close(rawpic);

// RGB Colour Thresholding-------------

selectWindow(RGBpic);

run("RGB Stack");

run("Convert Stack to Images");

selectWindow("Red");

rename("0");

selectWindow("Green");

rename("1");

selectWindow("Blue");

rename("2");

for (j=0;j<3;j++){

selectWindow(""+j);

setThreshold(low[j], high[j]);

setOption("BlackBackground", false);

run("Convert to Mask");

if (filter[j]=="stop")run("Invert");

}

imageCalculator("AND create", "0","1");

imageCalculator("AND create", "Result of 0","2");

for (j=0;j<3;j++){

selectWindow(""+j);

close();

}

selectWindow("Result of 0");

close();

selectWindow("Result of Result of 0");

rename("RGBmask");

run("Despeckle");

// RGB Colour Thresholding-------------

run("Images to Stack", "name="+rawpic+" title=[] use");

setSlice(2);

run("Invert", "slice");

setBatchMode(false);

setTool("freehand");

while (selectionType()==-1) {

waitForUser("Macro Paused", "Adjust the mask, select the ROI, then click OK to measure

area");

}

setBatchMode(true);

run("Make Inverse");

run("Fill", "stack");

run("Make Inverse");

setSlice(2);

run("Duplicate...", "title=temp");

selectWindow(rawpic);

setSlice(1);

run("Duplicate...", "title=ColourSample");

close(rawpic);

selectWindow("temp");

rename(rawpic);

run("Make Binary");

run("Set Measurements...", "area display redirect=None decimal=3");

run("Analyze Particles...", "summarize");

selectWindow("Summary");

lines = split(getInfo(), "\n");

values = split(lines[lengthOf(lines)-1], "\t");

setThreshold(255, 255);

run("Create Selection");

if (selectionType()==-1) {

print(rawpic+",0,N/A,N/A,N/A");

close("ColourSample");

close(rawpic);

}

else {

roiManager("add");

roiManager("select",(roiManager("count")-1));

roiManager("Rename", "Stain");

stain = roiManager("index");

close(rawpic);

selectWindow("ColourSample");

run("RGB Stack");

run("Convert Stack to Images");

selectWindow("Red");

roiManager("select", stain);

getStatistics(area, Rmean, min, max, std, histogram);

close("Red");

selectWindow("Green");

roiManager("select", stain);

getStatistics(area, Gmean, min, max, std, histogram);

close("Green");

selectWindow("Blue");

roiManager("select", stain);

getStatistics(area, Bmean, min, max, std, histogram);

close("Blue");

print(rawpic+","+values[2]+","+round(Rmean)+","+round(Gmean)+","+round(Bmean));

roiManager("Deselect");

roiManager("Delete");

}

//Image analysis

}

}

selectWindow("Log");

save(path+"/RGB-Threshold-Results.txt");

File.rename(path+"/RGB-Threshold-Results.txt", path+"/RGB-Threshold-Results.csv");

selectWindow("Summary");

run("Close");

selectWindow("Log");

run("Close");

setBatchMode(false);

exit("Macro Completed Successfully");
