# Supplementary material for: Damage-induced reactive oxygen species enable zebrafish tail regeneration by repositioning of Hedgehog expressing cells
Source: Nat Commun. 2018 Oct 1;9:4010. doi: 10.1038/s41467-018-06460-2 (PMC6167316; doi:10.1038/s41467-018-06460-2)
Supplement: Supplementary file 9 — Supplementary Software 5 [file 41467_2018_6460_MOESM9_ESM.docx]

Supplementary Software 5

Circumcircle

/*

* CALCULATING PROPERTIES OF A TRIANGLE'S CIRCUMCIRCLE

*

* Calculate the dimensions and radius of a circumcircle given three points on the circumference

Dave Mason [dnmason@liv.ac.uk] August 2017 Centre for Cell Imaging [http://cci.liv.ac.uk]

University of Liverpool Provided under a CCBY 4.0 Licence [https://creativecommons.org/licenses/by/4.0/]

*

*/

// close("*");

// newImage("Circumcircle", "8-bit black", 512, 512, 1);

//-- Collect three points

setTool("multipoint");

waitForUser("Select three points on the circle radius and hit OK");

getSelectionCoordinates(x, y);

run("Select None");

if (x.length != 3){

exit("Calculation requires only three points");

}

//-- First calculate the perimeter of the triangle

d1=sqrt((x[0]-x[1])*(x[0]-x[1])+(y[0]-y[1])*(y[0]-y[1]));

d2=sqrt((x[1]-x[2])*(x[1]-x[2])+(y[1]-y[2])*(y[1]-y[2]));

d3=sqrt((x[2]-x[0])*(x[2]-x[0])+(y[2]-y[0])*(y[2]-y[0]));

//-- Half the perimeter

s=0.5*(d1+d2+d3);

//-- Area of circumcircle

a=sqrt(s*(s-d1)*(s-d2)*(s*d3));

//-- Circumradius

r=(d1*d2*d3)/sqrt((d1+d2+d3)*(d2+d3-d1)*(d3+d1-d2)*(d1+d2-d3));

//-- Menger curvature is the inverse of the radius of the circle

mc=1/r;

//-- Centroid coordinates (from https://en.wikipedia.org/wiki/Circumscribed_circle#Cartesian_coordinates_2)

D=2*abs(x[0]*(y[1]-y[2]) + x[1]*(y[2]-y[0]) + x[2]*(y[0]-y[1]));

cenX=abs((x[0]*x[0]+y[0]*y[0]) * (y[1]-y[2]) + (x[1]*x[1]+y[1]*y[1]) * (y[2]-y[0]) + (x[2]*x[2]+y[2]*y[2]) * (y[0]-y[1]))/D;

cenY=abs((x[0]*x[0]+y[0]*y[0]) * (x[2]-x[1]) + (x[1]*x[1]+y[1]*y[1]) * (x[0]-x[2]) + (x[2]*x[2]+y[2]*y[2]) * (x[1]-x[0]))/D;

//-- report

print("Radius: "+r);

print("Menger Curvature: "+mc);

print(mc);

print("Circumcentre: "+cenX+","+cenY);

//-- Create an overlay

//-- Triangle

makePolygon(x[0],y[0],x[1],y[1],x[2],y[2]);

Overlay.addSelection("cyan");

//-- Circumcentre

makeLine(cenX-5,cenY,cenX+5,cenY);

Overlay.addSelection("magenta");

makeLine(cenX,cenY-5,cenX,cenY+5);

Overlay.addSelection("magenta");

//-- Circumcircle

run("Specify...", "width="+(r*2)+" height="+(r*2)+" x="+cenX+" y="+cenY+" oval centered");

Overlay.addSelection("red");

run("Select None");

exit("done");

}
